# Non-immune Hemolysis in Gaucher Disease and Review of the Literature

**DOI:** 10.5041/RMMJ.10446

**Published:** 2021-07-20

**Authors:** Eliyakim Hershkop, Idan Bergman, Alina Kurolap, Najib Dally, Hagit Baris Feldman

**Affiliations:** 1 The Ruth and Bruce Rappaport Faculty of Medicine, Technion–Israel Institute of Technology, Haifa, Israel; 2 Department of Internal Medicine, Maimonides Medical Center, Brooklyn, NY, USA; 3 The Genetics Institute and Genomics Center, Tel Aviv Sourasky Medical Center, Tel Aviv University, Tel Aviv, Israel; 4 The Hematology Unit, Ziv Medical Center, Safed, Israel; 5 The Azrieli Faculty of Medicine, Bar-Ilan University, Safed, Israel; 6 Sackler School of Medicine, Tel Aviv University, Tel Aviv, Israel

**Keywords:** Enzyme replacement therapy, Gaucher disease, hemolytic anemia, non-immune hemolytic anemia

## Abstract

Gaucher disease (GD) is an autosomal recessive disease characterized by the buildup of glucocerebrosides in macrophages, resulting in the formation of “Gaucher cells.” These cells predominantly infiltrate the liver, spleen, and bone marrow leading to hepatosplenomegaly, cytopenia, and bone pain. Anemia in GD is typically considered to result from non-hemolytic processes. Although rare, a higher rate of hemolytic anemia of the autoimmune type has been reported in GD than in the general population. The literature on non-immune hemolytic anemia in GD is scarce. We review the literature on hemolytic anemia in GD and report on a case of non-immune hemolytic anemia secondary to GD. We believe this is the first description of a patient with confirmed GD and symptomatic non-immune hemolytic anemia that responded to GD-specific treatment.

## INTRODUCTION

Gaucher disease (GD) is an autosomal recessive metabolic disease caused by pathogenic variants in the *GBA* gene, resulting in decreased activity of the enzyme glucocerebrosidase. Enzymatic partial activity or deficiency causes its substrate, glucocerebroside, to accumulate in cells, specifically in macrophages. These pathologic “Gaucher cells” often accumulate in the liver and spleen, invade the bone marrow, and are thought to be the cause of the typical symptoms of GD, i.e. hepatosplenomegaly, cytopenia, and bone pain.^1^

Gaucher disease has a broad phenotypic variation. Manifestations and symptoms range along a wide spectrum from completely asymptomatic to debilitating, fatal disease. Age of onset and diagnosis can also vary greatly from prenatal and neonatal to geriatric patients.^2^ Because of this great phenotypic variability, the progressive onset, and the rarity of the disease, patients with GD often experience a delay in diagnosis of 3–7 years on average.^2^,^3^ Patients with GD are often diagnosed incidentally due to low blood cell counts and/or splenomegaly, which are found on their annual checkups or during an acute illness.^4^ Thrombocytopenia is observed in 60%–90% of patients and is often severe, while anemia is seen in 20%–50% and is usually mild.^3^ Hemoglobin (Hb) levels may often be in the normal range even in the presence of severe thrombocytopenia. However, significant anemia with normal platelet counts is usually non-Gaucher related, suggesting an underlying comorbidity.^4^,^5^

Anemia refers to a decrease in red blood cells (RBCs), and is diagnosed in adults whose Hb values are lower than 13 g/dL in males or 12 g/dL in females. Anemia is typically caused by either RBC loss, RBC sequestration, decreased RBC production and/or increased RBC destruction, i.e. hemolysis—also referred to as hemolytic anemia (HA).^6^ There are two main categories of HA: (1) autoimmune hemolytic anemia (AIHA), in which RBC destruction is caused by antibodies; and (2) non-immune hemolytic anemia (NIHA), in which RBC destruction is caused by other factors, such as RBC structural defects leading to mechanical destruction.^7^

The cause of anemia in GD is likely multifactorial in nature, but it is most commonly described as non-hemolytic.^8^,^9^ The most common causes of anemia, as well as other cytopenias, in GD are thought to be due to hypersplenism, causing sequestration and destruction of cells in the spleen, and bone marrow infiltration by Gaucher cells causing impaired hematopoiesis.^3^,^8^ While there are reports of symptomatic HA in GD patients, these are most commonly of AIHA; reports of NIHA are extremely rare and have not been described in the literature of the past 30 years.^4^,^10^,^11^

We report on a patient with NIHA secondary to GD and review the available literature on hemolysis in GD patients. We believe this is the first description of a patient with confirmed GD, both genetically and biochemically, and symptomatic NIHA that responded to GD-specific treatment.

## CASE REPORT

A 35-year-old male of Ashkenazi Jewish descent presented to the Hematology Unit after a routine blood test revealed anemia, which appeared to be caused by a hemolytic process. His blood tests revealed a Hb level of 9.7 g/dL (normal range: 13–17), elevated reticulocyte count of 1.6×10^3^/mL (normal range: 0.03–0.09), low haptoglobin (<10 mg/dL, normal range: 30–200), and elevated lactase dehydrogenase (LDH) at 660 units/L (normal range: 125–220). His white blood cells (WBC) were within the normal range (4.3 mmol/L, normal range: 4–10.8), and he had mild thrombocytopenia (platelet count 137×10^3^/μL, normal range: 150–450). Bilirubin was not measured at the time, but three months earlier his total bilirubin was recorded as 1.9 mg/dL (normal range: 0.2–1.2), direct bilirubin 0.4 mg/dL (normal range: 0–0.3), and indirect bilirubin 1.5 mg/dL (normal range: 0–0.5). On blood test taken two months later, his iron was 62 μg/dL (normal range 65–175), transferrin 190 mg/dL (normal range 175–300), and ferritin 718 ng/mL (normal range: 30–300); of note, ferritinemia with normal iron and transferrin levels is a common feature in GD.^3^ Urobilinogen was not detected on urinalysis. His spleen size, measured by ultrasound, had increased over the past year from 14.5 cm to 15.7 cm. He also reported weight loss of approximately 6 kg over the past six months. He underwent an elective cholecystectomy two months earlier for symptomatic cholelithiasis, without postoperative complications. He was not taking any medications and denied recent infections.

Hematological workup revealed Coombs-negative HA, but the cause of the NIHA was not identified. A number of conditions were excluded, including hereditary spherocytosis, paroxysmal nocturnal hemoglobinuria, G6PD deficiency, and Wilson’s disease. In addition, an extensive Anemia–Immunology targeted gene panel 4.16 (Hematology Laboratory, Schneider Children’s Hospital, Petach Tikva, Israel) was negative for pathogenic variants (see supplementary data for full gene list). Screening for rheumatic processes returned negative. A bone marrow aspiration was performed, and Gaucher cells, as well as an increased erythroid precursor population, were visualized. No monoclonality, increase in blast population, or sign of myelodysplastic syndrome was detected. Gaucher disease was confirmed by low β-glucocerebrosidase activity (0.1 μmol/L/h, normal >2.5), and genetic testing revealed homozygosity for p.Asn409Ser (formerly known as N370S).

**Figure 1:**
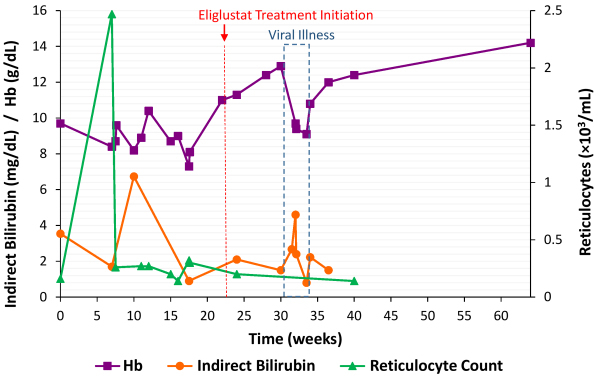
Patient Hemoglobin (Hb) (normal range: 13–17 g/dL), Indirect Bilirubin (normal range: 0.2–1.2 mg/dL), and Reticulocyte Counts (normal range: 0.03–0.09×10^3^/mL) Over Time. Gaucher disease treatment initiation with eliglustat and an incident of a viral illness are marked.

The patient’s clinical course over the subsequent months included two hospital admissions. Figure 1 presents the Hb and indirect bilirubin levels, as well as reticulocyte counts, observed over time. The first admission was due to jaundice, several days after a suspected viral gastroenteritis. The second admission was due to general weakness, an episode of pre-syncope, viral-like symptoms, and scleral icterus. The cause of the HA was not identified, and treatment with prednisone 60 mg daily was initiated, which was eventually tapered down to 5 mg once daily over the next several months. During this time, the patient also suffered from night sweats and exertional dyspnea. He underwent a full body CT scan that revealed hepatomegaly of 21 cm with an 8 mm hypodense lesion, splenomegaly of 17 cm, fractures of the 6th and 7th ribs on the right side, and bilateral infiltrates in the medulla of the femurs. The hepatosplenomegaly, fractured ribs, and infiltrated femurs were attributed to GD. Elective splenectomy was considered, and the patient received prophylactic vaccines against encapsulated bacteria.

Treatment for GD was initiated at our Gaucher clinic with the intent of treating the patient’s hepatosplenomegaly and bone disease, as well as to ameliorate the components of anemia and thrombocytopenia caused by GD. The patient received substrate reduction therapy (SRT) with eliglustat (Cerdelga™, Sanofi Genzyme, Cambridge, MA, USA), after efficacy and safety measures were ascertained. With treatment, the patient’s Hb and reticulocytosis significantly improved. Within three months, reticulocyte counts decreased to 0.14×10^3^/mL, his Hb increased steadily, reaching 14.2 g/dL, and total bilirubin decreased to 2.6 mg/dL. Since treatment initiation, there was a single occasion of deterioration in the patient’s cytopenia, which was attributed to a viral illness with quick recovery.

## DISCUSSION

Anemia is often multifactorial, which can make the underlying cause difficult to identify. Some of the main etiologies described include RBC loss, increased RBC destruction (hemolysis), decreased RBC production, and RBC sequestration.^6^,^12^

Hemolytic anemia is characterized by low Hb levels accompanied by jaundice, reticulocytosis, increased plasma LDH and indirect bilirubin levels, decreased plasma haptoglobin levels, and elevated urobilinogen in urine.^13^ It is distinguished as AIHA and NIHA; AIHA refers to RBC destruction by antibodies directed against erythrocyte antigens and is diagnosed by a positive Coombs test.^14^ In contrast, NIHA refers to RBC destruction in the absence of antibodies and is characterized by clinical signs of hemolysis and a negative Coombs test.^7^

The causes for anemia in GD are multifactorial in nature and can be categorized as either GD-related anemia or as anemia due to concurrent conditions, such as iron or vitamin B12 deficiency, myelodysplastic syndrome, or hematologic malignancies.^4^,^8^ The most significant contributors to anemia in GD are non-hemolytic, i.e. caused by sequestration and destruction of blood cells in the spleen, which is typically considered a non-hemolytic process,^3^,^7^,^8^ impaired hematopoiesis due to glucocerebroside infiltration of the bone marrow,^3^ iron dysregulation,^8^ and blood loss in patients with GD-related thrombocytopenia and thrombocytopathy.^5^

Hemolytic GD-related anemia is considered rare^11^; however, subclinical hemolysis may be common without patients expressing overt signs of RBC destruction, such as jaundice, increased bilirubin, and reticulocytosis.^15^–^17^ Suggested causes of HA include autoimmune antibodies,^8^ splenic and extra-splenic erythrophagocytosis,^15^,^17^–^19^ cytotoxicity of glucosphingosine,^8^,^20^ and non-immune intravascular hemolysis caused by fibrin shearing.^15^

Reports of symptomatic HA in GD with systemic and biological surrogate markers are rare and mostly of AIHA or HA of unknown type. Moreover, most publications provide only few details regarding the findings and clinical course of these patients, marking the lack of comprehensive knowledge on the role of GD diagnosis in HA manifestation. Table 1 summarizes all the published cases of HA in GD patients that we identified through a comprehensive literature review.

**Table 1:** Summary of Published Gaucher Disease Patients with Hemolytic Anemia

Year^REF^	Pt#*	Age (yr)	Sex	GD Dx; Genotype†	HSM	Bone Dis	Anemia (Hb, g/dL)	J	HBR	Retic	Other Anemia-related Features	Ab	HA Tx	Outcome
**Hemolytic Anemia of Unknown Type**														

**1942**^21^	1	41	M	GCs on spleen biopsy	Y	N	Y (~7.2 [45%])	Y	Y	Y	Myelogram characteristic of HA (active erythrogenesis)	n.a.	High caloric and vitamin diet, parenteral liver extract, iron, splenectomy	Improved

**1946**^17^	1	20	M	GCs on sternal puncture	N	Y	Y (12.5)	Y	Y	N	Abundant hemosiderin and active erythrophagia on sternal puncture	n.a.	n.a.	n.a.

**1954**^25^	2	42	M	GCs on either sternal marrow aspiration, splenic puncture, splenic tissue exam after splenectomy, or necropsy	Y	N	Y (11.9)	n.a.	Y	Y	n.a.	n.a.	n.a.	n.a.
	3	41	F		Y	N	Y (8.5)	n.a.	N	Y	n.a.	n.a.	n.a.	n.a.
	4	42	F		Y	N	Y (11.9)	n.a.	Y	n.a.	n.a.	n.a.	n.a.	n.a.
	6	43	F		Y	N	Y (10.3)	n.a.	Y	Y	n.a.	n.a.	n.a.	n.a.
	11	14	M		Y	N	Y (8.7)	n.a.	Y	n.a.	n.a.	n.a.	n.a.	n.a.
	15	12	M		Y	N	Y (10.6)	n.a.	Y	n.a.	n.a.	n.a.	Splenectomy	Died
	17	26	F		Y	N	Y (5.8)	n.a.	N	Y	n.a.	n.a.	Splenectomy	Died
	20	39	F		Y	N	Y (10.5)	n.a.	Y	Y	n.a.	n.a.	Splenectomy	n.a.
	25	19	F		n.a.	N	Y (11.0)	n.a.	Y	Y	n.a.	n.a.	Splenectomy	n.a.
	26	20	F		Y	n.a.	Y (8.0)	n.a.	n.a.	Y	n.a.	n.a.	Splenectomy	n.a.

**1960**^19^	1	n.a.	n.a.	GCs on BM aspirate	n.a.	n.a.	Y (11.5)	n.a.	n.a.	Y	Mild erythrophagocytosis	n.a.	n.a.	n.a.

**1968**^26^	2	29	M	GCs on liver biopsy and BM aspirate	Y	Y	Y (10.0)	N	Y	n.a.	Elevated urobilinogen	n.a.	n.a.	Died

**2008**^27^	1	2.5	F	Low β-GCase activity; p.[Asn409Ser]; [Ser395Phe]	Y	Y	Y	n.a.	n.a.	n.a.	n.a.	n.a.	Splenectomy	Died

**Autoimmune Hemolytic Anemia**														

**1955**^22^	29	n.a.	n.a.	n.a.	n.a.	n.a.	Y	n.a.	n.a.	n.a.	n.a.	Auto-Ab present	Blood transfusions, splenectomy, ACTH, and cortisone	Died

**1990**^10^	1	23	F	Low β-GCase activity	Y	n.a.	Y (4.0)	Y	Y	Y	Low haptoglobin, elevated LDH	Coombs +	Blood transfusions, steroids	Improved and stabilized
	2	n.a.	n.a.	n.a.	n.a.	n.a.	Y	n.a.	n.a.	n.a.	n.a.	Coombs +	n.a.	n.a.

**1994**^32^	2	37	F	Low β-GCase activity; p.[Asn409Ser]; [recTL]	Y	N	Y	n.a.	n.a.	n.a.	n.a.	n.a.	Blood transfusions, splenectomy	Resolved

**2002**^29^	1	n.a.	n.a.	n.a.	n.a.	n.a.	Y	n.a.	n.a.	n.a.	n.a.	n.a.	n.a.	n.a.
	2	n.a.	n.a.	n.a.	n.a.	n.a.	Y	n.a.	n.a.	n.a.	n.a.	n.a.	n.a.	n.a.

**2008**^27^	2	11 mo	M	Low β-GCase activity; p.[Thr102del]; [Thr102del]	Y	n.a.	Y	n.a.	n.a.	n.a.	n.a.	Coombs +	n.a.	Died

**2010**^31^	1	n.a.	F	n.a.	n.a.	n.a.	Y	n.a.	n.a.	n.a.	n.a.	Coombs +	n.a.	n.a.

**2014**^30^	1	n.a.	n.a.	n.a.	n.a.	n.a.	Y	n.a.	n.a.	n.a.	n.a.	n.a.	ERT	Died

**Non-immune Hemolytic Anemia**														

**1933**^23^	1	34	F	n.a.	Y	n.a.	Y (~4.0 [25%])	Y	Y	n.a.	Hemoglobinuria, nucleated RBCs, large pale cells and giant cells on spleen biopsy, bile pigment and salts intermittently present in urine	Negative auto-hemolysin	Blood transfusions, splenectomy	Improved

**2021 This study**	1	35	M	GCs on BM aspirate, low β-GCase activity; p.[Asn409Ser]; [Asn409Ser]	Y	Y	Y (9.7)	Y	Y	Y	Low haptoglobin, elevated LDH	Coombs-negative	SRT (eliglustat)	Full recovery

* Pt# refers to the patient number as published in the original manuscript (when available).

† Genotype according to new nomenclature (GBA NM_000157.4).

+, positive; Ab, antibodies; ACTH, adrenocorticotropic hormone; BM, bone marrow; Dx, diagnosis; ERT, enzyme replacement therapy; Dis, disease; F, female; GCs, Gaucher cells; GCase, glucocerebrosidase; GD, Gaucher disease; HA, hemolytic anemia; Hb, hemo-globin; HBR, hyperbilirubinemia; HSM, hepatospleno-megaly; J, jaundice; LDH, lactase dehydrogenase; M, male; mo, months; N, no; n.a., not available; RBCs, red blood cells; REF, reference; Retic, reticulocytosis; SRT, substrate reduction therapy; Tx, treatment; Y, yes; yr, years.

A number of reports of HA of unknown cause in GD have been published, most from the 1940s–1960s (Table 1).^10^,^17^,^19^,^21^–^27^ However, it is difficult to ascertain whether these cases were non-immune or immune-mediated, as some of them preceded Coombs testing, which was first described in 1945,^28^ and those published after that date make no mention of using the test. Additionally, the diagnosis of GD in many of these cases was based solely on the visualization of Gaucher cells, which can be difficult to distinguish from pseudo-Gaucher cells present in other diseases.^3^

Wasserman et al. (writing in 1955) were the first to report of Coombs-positive hemolytic anemia in GD,^22^ which was followed by several case series consistent with a 0.55%–2.7% rate of AIHA in GD patients^10^,^27^,^29^–^31^ and several additional cases (Table 1).^4^,^32^ Autoimmune hemolytic anemia (AIHA) is reported to be more common in GD patients than in the general population, which has an annual incidence of 1 in 100,000 (0.001%).^4^,^33^ This may be explained by the elevated antibody levels present in GD patients^4^,^10^,^34^; however, in 2018 Serratrice et al. demonstrated that the higher incidence of antibodies in GD does not necessarily translate into an increased incidence of clinical autoimmune diseases.^35^

In 1933, Carling et al. provided the only published case of NIHA with confirmed absence of autoantibodies in a GD patient; however, the authors did not specify how GD was diagnosed.^23^ They reported on a 34-year-old female with fever, vomiting, jaundice, hepatomegaly, and a 16-year history of 3–4 annual “blood crises” with jaundice, indirect bilirubinemia, hemoglobinuria, epigastric pain, and transient splenomegaly. She had 25% hemoglobin (approximately 4 g/dL)^36^ and nucleated RBCs. Autohemolysin was not detected with either warm or cold conditions when her serum and washed cells were mixed, consistent with NIHA. She was treated by splenectomy with improvement.^22^,^23^

Anemia due to GD typically improves with treatment using enzyme replacement therapy (ERT) within 3–6 months and plateaus within 18–36 months^4^; eliglustat, a SRT, has also been shown to be effective.^37^ In the event that the anemia does not resolve, one can suspect that it resulted from a non-GD etiology and should be further investigated.^8^ The recommended treatment for AIHA is steroids,^4^ and it is not expected to improve with GD-specific therapy, while NIHA treatments vary based on the etiology.^13^ Since eliglustat therapy completely resolved the NIHA in the patient we describe, it is plausible to assume that his hemolytic process was strictly GD-related.

## CONCLUSION

In summary, our report presents a patient with significant anemia but mild thrombocytopenia, not consistent with severe GD.^4^,^5^ Although GD was not thought to be the main cause of this patient’s anemia, in view of his dramatic response to SRT and lack of any other etiology for his NIHA, it seems that it can be attributed exclusively to GD. To our knowledge, this is the first report of two aspects: first, this is the first documented patient with GD, confirmed both genetically and biochemically, who had Coombs-negative symptomatic HA; and second, it is the first report of GD with symptomatic NIHA that resolved with GD-specific treatment. Our report emphasizes the importance of considering GD in the differential diagnosis when treating NIHA, even when severe thrombocytopenia is not present. This knowledge can be invaluable for clinicians to help guide their treatment decision-making, supporting prompt initiation of GD treatment, while simultaneously excluding other causes of NIHA.

## Supplementary Information



## Abbreviations

AIHA: autoimmune hemolytic anemia
CT: computed tomography
ERT: enzyme replacement therapy
GD: Gaucher disease
HA: hemolytic anemia
Hb: hemoglobin
LDH: lactase dehydrogenase
NIHA: non-immune hemolytic anemia
RBC: red blood cells
SRT: substrate reduction therapy
WBC: white blood cells
