# Supplementary material for: Non-immune Hemolysis in Gaucher Disease and Review of the Literature
Source: Rambam Maimonides Med J. 2021 Jul 20;12(3):e0025. doi: 10.5041/RMMJ.10446 (PMC8284991; doi:10.5041/RMMJ.10446)
Supplement: Supplementary file 1 [file rmmj-12-3-e0025-AM.pdf]

SUPPLEMENTARY MATERIAL

This appendix has been provided by the authors for the benefit of readers

Supplement to Non-immune Hemolysis in Gaucher Disease and Review of the Literature

Hershkop E, Bergman I, Kurolap A, Dally N, Baris Feldman H. Non-immune Hemolysis in Gaucher Disease and Review of the Literature. Rambam Maimonides Med J 2020;12 (3):e0025. doi:10.5041/RMMJ.10446

LIST OF GENES IN THE ANEMIA-IMMUNOLOGY TARGETED GENE PANEL 4.16:

ABCB6, ABCB7, ADA2, AICDA, AK1, ALAS2, ALDOA, AMN, ANK1, AP3B1, ATN1, ATRX B2M, BCORL1, BHLHE41, BPGM, BTK C15ORF41, CARD9, CARD11, CD19, CD20, CD3D, CD3e, CD3g, CD3z, CD27, CD40, CD40L CDAN1, CECR1, CIITA, CORO1A,CP, CPO, CSMD1, CYBA, CTLA4, CYB5R3, CYBB,CUBN, DOCK2, DOCK8, EGLN1, EGLN2, EGLN3, EPAS1, EPB41, EPB42, EPO, EPOR, FAS, FASL, FAM135A, FINB, FOXP3, GCLC, G6PD, GATA1, GATA2, GFI1B, GLRX5, GNL3, GSR,GSS, HFE, HIF1A, HIF3A, HIF1AN, HJV, HK1, HSPA9, ICOS, IFNG, IFNGR1, IFNGR2, IL10, IL10RA, IL12B, IL17F, IL17RA, IL21, IL23A, IL2RA, IL2RG, IL7R, INO80, ITPKC, ITPR3, IRF8, ISG15, ITCH, ITK, JAK2, JAK3, KCNN4, KDM6A, KLF1, KNSL5, KRAS, LCK , LRBA, LRRC3, LYST, MACF1, MAGT1, MCM2, MDC1, MOGS, MSH6, MST1, Munc13-4, MYD88, NCF1, NCF2, NCF4, NFKB2, NDUFB11, NEMO, NRAS, NT5C3A, ORAi1, OS9, PARP4, PGK1, Piezo1, PIGA, PKLR, PIK3CD, PIK3R1, PPO, PRF1, RAG1, RAG2, RhAG, PRKCD, RFX5, RFXANK, RFXAP, RPL15, RPL18, RPL23, RPL11, RPL27, RPL31, RPL5, RPS10, RPS14, RPS17, RPS24, RPS26, RPS27, RPS28 RPS7, RPS8, RPL35, RPL36, RPS15, RPS18, RPS27A, RTL1, SH2B3, SLC24A2, SLC2A12, SEC23B, SH2D1A, SLC11A2, SLC19A2, SLC25A38, SLC2A1, SLC40A1, SLC4A1, SLC7A7, SPTA1, SPTB, STAT1, STAT3, STEAP3, STIM1, STXBP2, TACI, TAP1, TAP2, TAPBP, TNFRSF12, TNFRSF6, TMPRSS6, TPI1, TPP2, TSR2, TYK2, TRNA-SER UMPH1, UNC13D, UNG, UROS, WDR86, ZNF197, ZXDC; 95% coverage: CASP10, CASP8, XIAP, ITK, MAGT1, PLDN, RPS8, RPL26, RPL35A, RPS19, RPS29, TF, HAMP, TFRC2, YARS2, TRNT1, TSR2, GPI, CD59, VHL, SF3B1, FECH, AIRE, IL10RB, PUS1, RAB27A, STX11 UGT1A1. RNA only: TRS2:chr6:27495814-895
